# Amelioration of Hyperglycemia-Induced Nephropathy by 3,3′-Diindolylmethane in Diabetic Mice

**DOI:** 10.3390/molecules24244474

**Published:** 2019-12-06

**Authors:** Kyeong-Mi Choi, Hwan-Soo Yoo

**Affiliations:** College of Pharmacy, Chungbuk National University, Osongsaengmyeong 1-ro, Heungduk-gu, Cheongju 28160, Korea; mirine0101@hanmail.net

**Keywords:** 3,3′-diindolylmethane, hyperglycemia, nephropathy, PKC-α, TGF-β1

## Abstract

Type 1 diabetes mellitus (insulin-dependent diabetes) is characterized by hyperglycemia caused by an insulin deficiency. Diabetic nephropathy is a major complication of hyperglycemia. 3,3′-diindolylmethane (DIM)-a natural compound produced from indole-3-carbinol, found in cruciferous vegetables-enhances glucose uptake by increasing the activation of the insulin signaling pathway in 3T3-L1 adipocytes. In this study, we investigated whether DIM could improve insulin-dependent diabetes and nephropathy in streptozotocin (STZ)-induced diabetic mice. In mice, STZ induced hyperglycemia, hunger, thirst, and abnormally increased kidney weight and serum creatinine, which is a renal functional parameter. DIM decreased STZ-increased high blood glucose levels and food and water intake in diabetic mice. DIM also improved diabetic nephropathy by inhibiting the expression of PKC-α, the marker of albuminuria, and TGF-β1, an indicator of renal hypertrophy, in diabetic mice. Our findings suggest that DIM may ameliorate hyperglycemia and diabetic nephropathy through the inhibition of PKC-α and TGF-β1 signaling.

## 1. Introduction

Type 1 diabetes mellitus is a chronic disease, which is characterized by insulin deficiency due to the loss of pancreatic β cells and the resultant abnormally high blood glucose [1,2]. Diabetic nephropathy is a major chronic complication, caused by uncontrolled hyperglycemia in type 1 diabetes [3,4]. Pathological changes, such as glomerular hypertrophy, mesangial cell expansion, and tubulointerstitial fibrosis, occur during the progression of diabetic nephropathy [4,5].

The pathogenesis of diabetic nephropathy eventually triggers albuminuria and a decrease in the glomerular filtration rate, and is associated with complex molecular mechanisms, including those of protein kinase C (PKC) and transforming growth factor-β1 (TGF-β1) [4]. PKC is a family of serine threonine kinases, including PKC-α, -β1, -β2, -δ, and -ε, and is abnormally activated in the development of diabetic nephropathy [4,6]. The overexpression of PKC-α causes albuminuria and oxidative stress during the course of diabetic nephropathy [4,7]. TGF-β1 is upregulated in the progression of diabetic nephropathy and plays an important role in the induction of renal hypertrophy [4,8,9]. TGF-β1 has also been known to promote renal fibrosis in diabetic nephropathy through the activation of Smad and mitogen-activated protein kinase (MAPK), including p38 MAPK, c-Jun *N*-terminal kinase, and extracellular signal-regulated kinase signaling pathways [4,9,10].

3,3′-diindolylmethane (DIM) is a natural compound produced from the acid-catalyzed self-condensation of indole-3-carbinol, which is abundant in cruciferous vegetables, such as broccoli and cabbage [11,12]. Previous studies have found that DIM can improve type 2 diabetes by enhancing glucose uptake through the activation of insulin signaling in 3T3-L1 cells, and by lowering the plasma glucose levels in high-fat-diet-fed obese mice [13,14]. 

Streptozotocin (STZ) is widely used to study the pathology of diabetes mellitus and diabetic complications in most strains of rodents [15]. STZ is the most prominent diabetogenic chemical—it is toxic to the insulin-producing pancreatic β cells, and damages the kidney and liver tissues [15,16,17,18,19]. In this study, our goal was to determine whether DIM could improve STZ-induced diabetes and nephropathy in mice, as well as to elucidate its underlying mechanism. 

## 2. Results

### 2.1. DIM Improves Blood Glucose Levels and Food and Water Intakes in Diabetic Mice 

**Figure 1:**
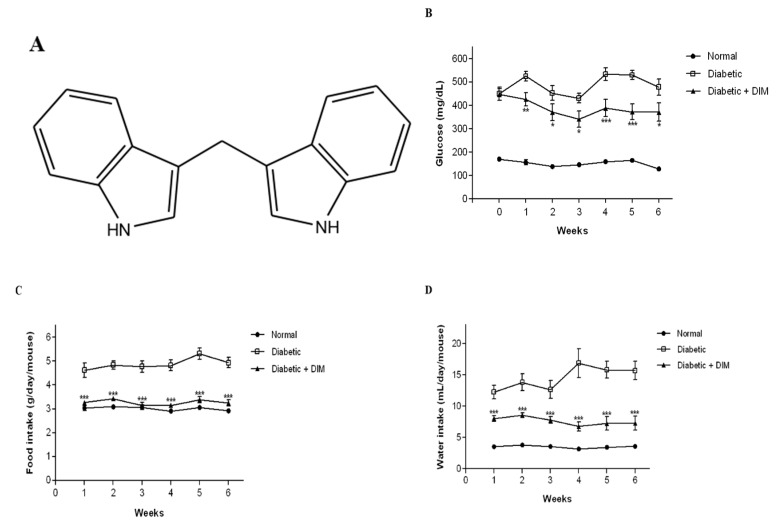
The inhibitory effects of 3,3′-diindolylmethane (DIM) on hyperglycemia in diabetic mice. (**A**) Chemical structure of DIM. The STZ-induced diabetic mice were fed a diet with or without DIM for 6 weeks. (**B**) The fasting-blood glucose level was measured weekly. (**C**) Food and (**D**) water intake were recorded four times per week and twice a week, respectively. Values are expressed as mean ± SE (n = 10). * *p* < 0.05, ** *p* < 0.01, *** *p* < 0.001, significantly different from the diabetic group.

We examined whether DIM (Figure 1A) ameliorates STZ-induced diabetes. The levels of fasting-blood glucose and the body weights of the mice were measured once a week. The blood glucose levels of STZ-induced diabetic mice were apparently increased compared with those of normal mice (Figure 1B). Blood glucose levels in normal and diabetic groups at 0 weeks were approximately 170.8 ± 7.4 and 448.9 ± 26.3 mg/dL, respectively. DIM-treated mice showed that the blood glucose levels in the first week were decreased by approximately 18.9% compared to diabetic mice. The decreased levels of blood glucose in the diabetic plus DIM-treated group were maintained throughout the experimental period and ranged within 18–30%. During the experimental period, the mean blood glucose levels in the normal-, diabetic-, or diabetic plus DIM-treated groups were approximately 152.4 ± 6.9, 486.3 ± 26.3, or 388.6 ± 33.5 mg/dL, respectively. 

Hyperglycemia caused excessive hunger and thirst. Food intake was measured four times per week, and water intake was measured twice a week. The food and water intakes of STZ-induced diabetic mice were significantly increased compared with those of normal mice (Figure 1C,D). Food intakes in the normal-, diabetic-, or diabetic plus DIM-treated mice at the first week were approximately 3.0 ± 0.1, 4.6 ± 0.3, or 3.3 ± 0.0 g/day/mouse, respectively. DIM significantly decreased the diabetic-mediated increase in food intake in the diabetic plus DIM group, to a level similar to that of the normal mice throughout the experimental period. The mean food intakes in the normal-, diabetic-, or diabetic plus DIM-treated groups during the experimental period were approximately 3.0 ± 0.1, 4.9 ± 0.2, or 3.3 ± 0.1 g/day/mouse, respectively. DIM also significantly reduced water intake in the diabetic plus DIM-treated group compared to that of the diabetic mice. The water intakes result in the normal-, diabetic-, or diabetic plus DIM-treated mice at the first week were approximately 3.5 ± 0.1, 12.3 ± 1.1, or 8.0 ± 0.5 mL/day/mouse, respectively. The decreased water intake in the diabetic plus DIM-treated group was maintained over the experimental period at the range of 35%–60%. During the experimental period, the mean water intakes result in the normal-, diabetic-, or diabetic plus DIM-treated group were approximately 3.5 ± 0.2, 14.5 ± 1.5, or 7.6 ± 0.7 mL/day/mouse, respectively. These results suggested that DIM improved STZ-induced hyperglycemia, hunger, and thirst.

### 2.2. DIM Inhibits Hyperglycemia-Induced Kidney Damage of Diabetic Mice 

**Figure 2:**
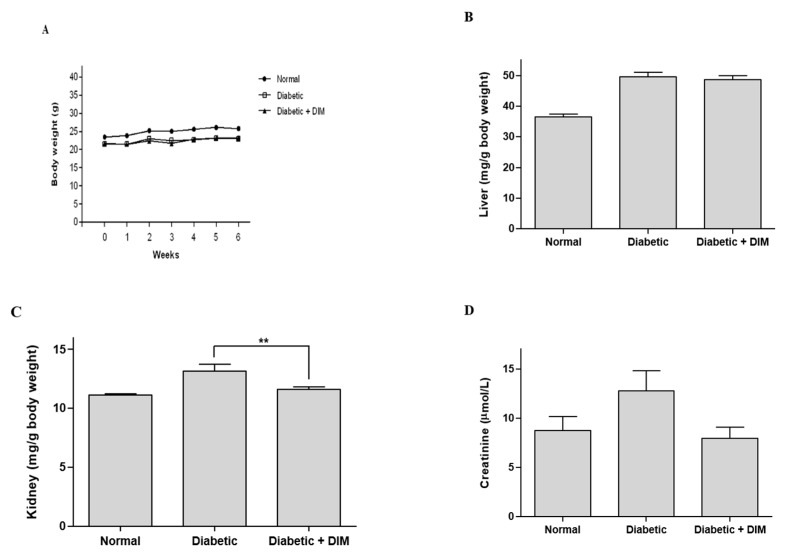
The inhibitory effect of DIM on hyperglycemia-induced renal toxicity in diabetic mice. (**A**) The body weight was measured weekly. (**B**) The livers and (**C**) kidneys were obtained from mice and weighed after mice fasted for 15 h at the end of the study. (**D**) The serum was collected from the mice and the creatinine level was measured. Values are expressed as mean ± SE (n = 10). ** *p* < 0.01, significantly different from the diabetic group.

Hyperglycemia leads to weight loss and nephropathy. The body weights of diabetic mice were decreased over 6 weeks after STZ administration, compared with normal mice (Figure 2A). Liver weights in STZ-induced diabetic mice were significantly higher than those of normal mice by approximately 26.3% (Figure 2B). However, DIM did not exhibit any change in the body and liver weights between diabetic and diabetic plus DIM-treated groups. The kidney weights of the diabetic mice were increased by approximately 15.7% compared to those of normal mice (Figure 2C). DIM significantly lowered the increased kidney weights in diabetic mice by approximately 12.1%.

Serum creatinine is a biomarker for kidney function. Serum creatinine levels in diabetic mice were increased by approximately 31.5% compared to those in normal mice (Figure 2D). However, DIM decreased the diabetic-mediated increase in creatinine by approximately 37.9% compared to that in diabetic mice. These results suggested that DIM may restore kidney function in STZ-induced diabetic mice.

### 2.3. DIM Inhibits Hyperglycemia-Induced Activation of Pkc-α and Tgf-β1 in the Kidneys 

**Figure 3:**
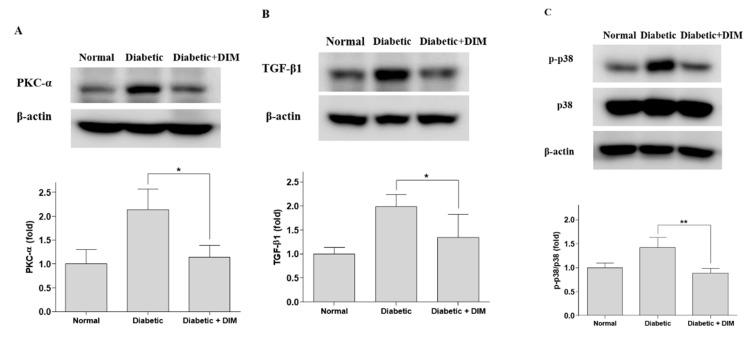
Decreased expression of PKC-α, TGF-β1, and p-p38 by DIM in the kidney tissues of mice. The kidneys were homogenized and lysed followed by western blot analysis for (**A**) PKC-α, (**B**) TGF-β1, (**C**) p-p38. Values are expressed as mean ± SE. * *p* < 0.05, ** *p* < 0.01, significantly different from the diabetic group.

Hyperglycemia induces an abnormal activation of the PKC and TGF-β pathways involved in the pathogenesis of diabetic nephropathy. The expression of PKC-α, which is associated with albuminuria in diabetic nephropathy, was significantly increased in the kidney tissues of STZ-induced diabetic mice, by approximately 113.8% compared with that of normal mice (Figure 3A). DIM strongly inhibited the increased PKC-α expression in diabetic mice by approximately 46.7%. The expression of TGF-β1, which plays an important role in kidney hypertrophy and fibrosis, was significantly elevated in the kidney tissues of STZ-induced diabetic mice by approximately 98.9% compared with that of normal mice (Figure 3B). DIM significantly inhibited the increased TGF-β1 expression in diabetic mice by approximately 32.5%.

**Figure 4:**
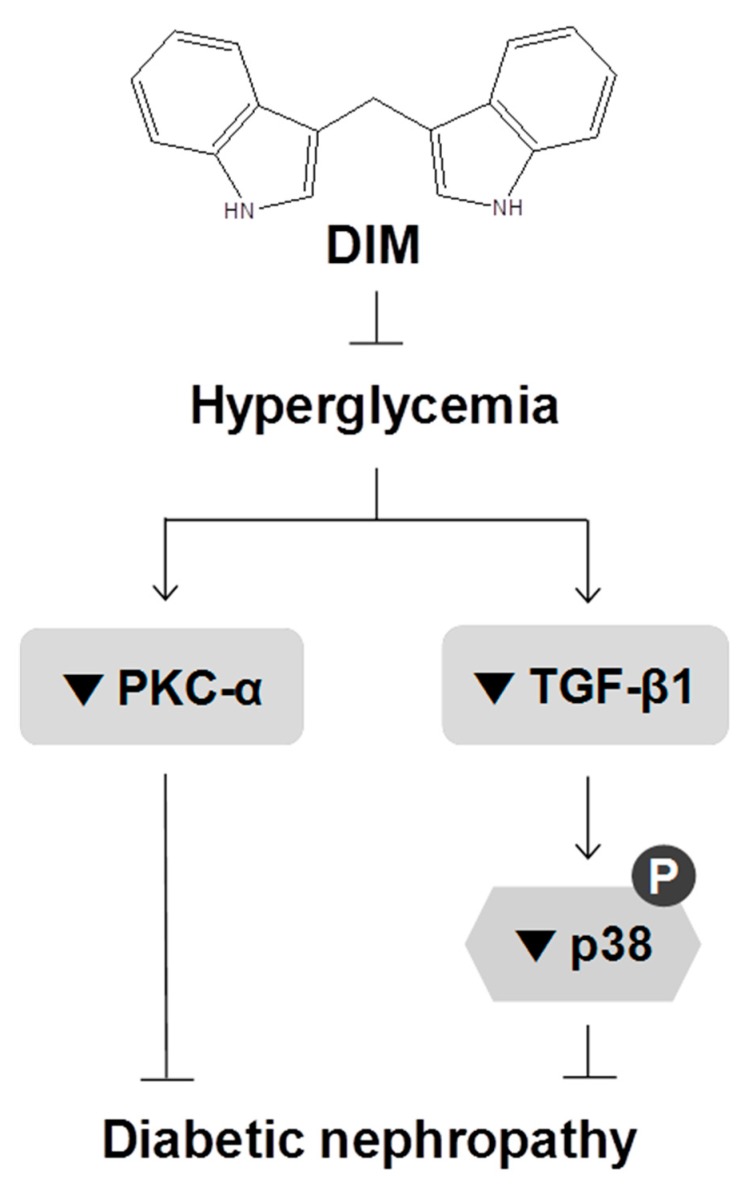
The proposed mechanism whereby DIM ameliorates hyperglycemia and diabetic nephropathy. Our data suggest that DIM protects diabetic mice from kidney damage induced by hyperglycemia through the inhibition of PKC-α and TGF-β1 signaling. The symbols ⊥, ↓, and ▼ represent inhibition, stimulation, and downregulation, respectively.

p38 MAPK is a downstream signaling molecule in the TGF-β pathway in the pathogenesis of diabetic nephropathy. The phosphorylation of p38 was significantly increased in the kidney tissues of STZ-induced diabetic mice, by approximately 42.1% compared with that of normal mice (Figure 3C). DIM markedly inhibited the increased p38 phosphorylation in the diabetic group by approximately 37.5%. These results suggested that DIM may ameliorate hyperglycemia-induced diabetic nephropathy through the suppression of PKC-α and TGF-β1 signaling (Figure 4).

## 3. Discussion

Type 1 diabetes mellitus is a metabolic disease resulting from the destruction of insulin-producing β cells in the pancreas, that leads to hyperglycemia [1,2,20]. DIM, a major metabolite of indole-3-carbinol, which is naturally produced in broccoli and cabbage, enhances glucose uptake through the improvement of insulin sensitivity in 3T3-L1 cells [13]. STZ has been widely used to induce type 1 diabetes in experimental animals by causing an abnormality in the β cell function of the pancreatic islets [15,16,17]. Thus, we investigated whether DIM could improve STZ-induced diabetes in mice and elucidated its underlying mechanism.

Type 1 diabetes is characterized by hyperglycemia, thirst, hunger, and weight loss, which are caused by insulin deficiency [1,2]. In this study, the blood glucose levels, and food and water intakes were strongly increased, and body weights were decreased in the STZ-induced diabetic mice. DIM markedly lowered the blood glucose levels in STZ-induced diabetic mice. Similarly, DIM decreased the plasma glucose levels in high-fat-diet-fed obese mice, and red cabbage extract lowered the blood glucose levels in STZ-treated rats [14,21]. Broccoli sprouts reduced the fasting blood glucose and serum insulin levels in type 2 diabetic patients [22,23]. In the present study, DIM also strongly reduced the food and water intakes of STZ-induced diabetic mice. In particular, diabetic plus DIM-treated mice showed a similar pattern of food intakes to those of the normal mice. Thus, DIM may improve the hunger and thirst of diabetic mice by inhibiting hyperglycemia.

Hyperglycemia is a major risk factor in the development of diabetic nephropathy [2,4,6]. We found that the kidney weights were increased following hyperglycemia in diabetic mice and restored after DIM treatment. These results suggest that DIM ameliorates hyperglycemia-induced renal hypertrophy. In this study, DIM also reduced high serum creatinine levels—an indicator of renal dysfunction, induced by hyperglycemia in diabetic mice. Similarly, DIM decreased serum creatinine and blood urea nitrogen levels in lipopolysaccharide-induced acute kidney injury mice [24].

Hyperglycemia mediates an abnormal activation of PKC-α and TGF-β1, which play an important role in the development of diabetic nephropathy [4,6,25]. PKC-α is activated in the kidneys of STZ-induced hyperglycemic mice, and PKC-α-deficient diabetic mice were protected from albuminuria [4,26]. In this study, DIM inhibited the hyperglycemia-induced activation of PKC-α in the kidney tissues of diabetic mice, indicating that DIM may protect diabetic mice from albuminuria by lowering PKC-α overexpression. TGF-β1 expression was increased in the kidneys of diabetic mice, rats, and humans, and the neutralization of TGF-β in STZ-induced diabetic mice attenuated renal hypertrophy [8,27]. TGF-β1 also promoted renal fibrosis via the activation of signaling pathways, such as p38 MAPK and Smad in mice and human kidney diseases [4,9]. DIM ameliorated renal fibrosis in mice with unilateral ureteral obstruction via the inhibition of the activation of TGF-β1 signaling [28]. We found that DIM decreased the elevated expression of TGF-β1 and p-p38 in the kidney tissues of diabetic mice, indicating that DIM may improve renal hypertrophy and fibrosis in diabetic mice through the inhibition of TGF-β1 signaling. These results suggest that DIM may improve hyperglycemia-mediated kidney damage by lowering PKC-α and TGF-β1 signaling in diabetic mice.

In conclusion, we present evidence that DIM ameliorates hyperglycemia and diabetic nephropathy through the downregulation of PKC-α and TGF-β1 signaling in diabetic mice. Thus, DIM may be a potential therapeutic agent for improving hyperglycemia and diabetic nephropathy.

## 4. Materials and Methods

### 4.1. Materials

DIM, STZ, sodium citrate, citric acid, and a creatinine assay kit were purchased from Sigma–Aldrich Co. LLC. (Saint Louis, MO, USA). Bovine serum albumin (BSA) was obtained from Roche Diagnostics (Mannheim, Germany). A Pierce bicinchoninic acid (BCA) protein assay kit was obtained from Thermo Fisher Scientific (Waltham, MA, USA). RIPA lysis buffer, and protease and phosphatase inhibitors were purchased from Atto Corp. (Tokyo, Japan). Antibodies against PKC-α, p38 MAP kinase (p38), and phospho-p38 MAP Kinase^Thr180/Tyr182^ (p-p38) were obtained from Cell Signaling Technology (Beverly, MA, USA). The antibody against TGF-β1 was obtained from Abcam, PLC. (Cambridge, MA, USA). The antibody against β-actin was purchased from Santa Cruz Biotechnology, Inc. (Santa Cruz, CA, USA). All chemicals were of analytical grade.

### 4.2. Animal Treatment 

The study protocol was approved by the Animal Care and Use Committee of Chungbuk National University. Male 8-week-old C57BL/6J mice were purchased from Japan SLC, Inc. (Shizuoka, Japan). All the mice were housed in a room with controlled temperature (21–23 °C), humidity (55–60%), and lighting (12 h light/dark cycle), and were supplied with water and a basal diet ad libitum. Mice were acclimated for 1 week, followed by fasting for 18 h, then diabetes was induced in the mice by a single intraperitoneal injection of 100 mg/kg body weight of STZ (freshly dissolved in 0.05 M sodium citrate buffer, pH 4.5). Normal mice were administrated the citrate buffer. After 1 week, mice with 6 h fasting-blood glucose levels exceeding 300 mg/dL were considered as diabetic. The STZ-induced diabetic mice were randomly divided into a diabetic group (n = 10) and a diabetic plus DIM-treated group (n = 10). The diabetic mice were fed a LAB Rodent Chow diet (DongAone Corp. Ltd.; Dangjin, Korea) with or without 0.5% DIM (5 g DIM/kg diet) for 6 weeks. The normal mice (n = 10) were fed a LAB Rodent Chow diet. During the experiment, blood was collected from the tail vein of the mice, and the 6 h fasting-blood glucose level was measured. At the end of the experimental period, the mice were anesthetized, and blood was collected. The kidneys and livers were excised, rinsed with saline solution, weighed, and stored at −70 °C.

### 4.3. Creatinine Assay

All the mice were anesthetized using diethyl ether after 15 h of fasting. Blood was collected from the abdominal vena cava and centrifuged at 3500 *g* for 10 min at 4 °C. Serum creatinine concentration was determined using a creatinine assay kit. Creatinine was converted to sarcosine enzymatically and quantified with a Molecular Devices ELISA reader at 570 nm.

### 4.4. Western Blot Analysis

The kidneys were homogenized in RIPA lysis buffer containing protease and phosphatase inhibitors. The homogenates were centrifuged at 18,300 *g* for 30 min at 4 °C, and the supernatant was collected. The total protein concentration of the lysates was determined using a BCA protein assay kit. Proteins in the lysates were separated through 10% sodium dodecyl sulfate-polyacrylamide gel electrophoresis (SDS-PAGE) and then transferred to polyvinylidene difluoride (PVDF) membranes (Merck Millipore Ltd., Darmstadt, Germany). The membranes were blocked with 5% BSA overnight at 4 °C, then incubated overnight at 4 °C with the following primary antibodies: PKC-α, TGF-β1, p38, p-p38, or β-actin. The membranes were incubated with horseradish peroxidase (HRP)-conjugated secondary antibodies overnight at 4 °C. Specific bands were visualized using enhanced chemiluminescence (ECL) detection reagents for western blots (GE Healthcare Life Sciences, Buckinghamshire, United Kingdom) and analyzed with a chemiluminescence imaging system (Amersham Imager 600; GE Healthcare Bio-Sciences AB, Uppsala, Sweden).

### 4.5. Statistical Analysis

All values are expressed as the mean ± standard error (SE). Statistical significance was determined using a one-way analysis of variance, followed by the Newman–Keuls Multiple Comparison test. *p* < 0.05 was considered statistically significant.
